# Estimates of Seasonal Influenza Burden That Could Be Averted by Improved Influenza Vaccines in the Australian Population Aged Under 65 Years, 2015–2019

**DOI:** 10.1111/irv.13289

**Published:** 2024-04-18

**Authors:** Alicia N. Stein, Catherine G. A. Pendrey, David J. Muscatello, Paul G. Van Buynder, James E. Fielding, Jason C. Menche, Sheena G. Sullivan

**Affiliations:** ^1^ CSL Seqirus Medical Affairs Melbourne Australia; ^2^ WHO Collaborating Centre for Reference and Research on Influenza Royal Melbourne Hospital, at the Peter Doherty Institute for Infection and Immunity Melbourne Australia; ^3^ National Centre for Epidemiology and Population Health Australian National University Canberra Australia; ^4^ School of Population Health University of NSW Kensington Australia; ^5^ School of Medicine Griffith University Southport Australia; ^6^ Victorian Infectious Diseases Reference Laboratory at the Peter Doherty Institute for Infection and Immunity Melbourne Australia; ^7^ Department of Infectious Diseases University of Melbourne, at the Peter Doherty Institute for Infection and Immunity Melbourne Australia; ^8^ Department of Epidemiology University of California, Los Angeles Los Angeles California USA

**Keywords:** burden of disease, influenza, influenza vaccines, vaccine effectiveness

## Abstract

**Background:**

The interpretation of relative vaccine effectiveness (rVE) of improved influenza vaccines is complex. Estimation of burden averted is useful to contextualise their potential impact across different seasons. For the population aged under 65 years in Australia, this study estimated the additional morbidity and mortality that could be averted using improved influenza vaccines.

**Methods:**

We used observed, season‐specific (2015–2019) influenza notification and influenza‐coded hospitalisation frequencies and published modelled estimates of influenza‐associated hospitalisations and deaths that occurred under the prevailing influenza vaccination coverage scenario. After back‐calculating to the estimated burden in the population without vaccination, we applied published standard influenza vaccine effectiveness and coverage estimates to calculate the burden potentially averted by standard and improved influenza vaccines. A plausible range of rVE values were used, assuming 50% coverage.

**Results:**

The percentage point difference in absolute vaccine effectiveness (VE) of an improved vaccine compared to a standard vaccine is directly proportional to its rVE and inversely proportional to the effectiveness of the standard vaccine. The incremental burden averted by an improved vaccine is a function of both its difference in absolute VE and the severity of the influenza season. Assuming an rVE of 15% with 50% coverage, the improved vaccine was estimated to additionally avert 1517 to 12,641 influenza notifications, 287 to 1311 influenza‐coded hospitalisations and 9 to 33 modelled all‐cause influenza deaths per year compared to the standard vaccine.

**Conclusions:**

Improved vaccines can have substantial clinical and population impact, particularly when the effectiveness of standard vaccines is low, and burden is high.

## 1 Introduction

Seasonal influenza has been estimated to annually cause 290,000–650,000 respiratory deaths globally [1]. In Australia, influenza accounts for an estimated 1000 respiratory and 2800 all cause deaths [2] and 21,000 hospitalisations [3], annually.

Vaccination is the primary strategy to reduce influenza transmission and disease burden [4, 5, 6]. Standard inactivated egg‐based vaccines are manufactured by combining the haemagglutinin (HA) and neuraminidase (NA) genes from selected variants with genes from viruses designed to grow well in eggs [7]. Standard vaccines have various limitations and a range of alternative vaccine technologies have been developed. High‐dose egg‐based vaccines contain a fourfold increase of the HA antigen to increase immunogenicity [4, 7]. Adjuvanted egg‐based vaccines include an adjuvant that enhances the immune response to the vaccine antigen [5]. Cell‐based vaccines propagate vaccine viruses in cell culture to avoid the risk of egg adaptations that alter antigenicity and compromise vaccine effectiveness (VE) [6]. Recombinant vaccines similarly avoid problems of egg adaptation by using a recombinant technology to produce purified HA from a baculovirus overexpression system. In all cases, the vaccines stimulate antibody recognition of the HA protein. Ongoing advances in vaccine technology, including the development of mRNA‐based influenza vaccines, have the potential to produce additional improved vaccines [8].

Frequent mutation in the HA enables the virus to escape antibody recognition and requires frequent updates to the recommended composition of influenza vaccines. Updated recommendations are issued by the World Health Organization each February for the northern hemisphere and each September for the southern hemisphere [7]. Both frequent mutation and frequent updates to vaccine composition mean that influenza VE varies annually depending on the degree of antigenic similarity between the vaccine and circulating viruses.

Multiple other factors also influence VE, including the level of influenza activity, the recipient's immune response and health status, and population demographics and pre‐existing immunity [7, 9, 10]. When different types of vaccines are compared, the incremental benefits of one vaccine over another (relative VE or rVE) may appear small. However, VE and rVE do not clearly communicate the benefits of an improved vaccine at a population level, and marginal differences in rVE may represent large‐scale differences in the absolute numbers of hospitalisations and lives saved [9, 11, 12].

Evaluating the population health impact of vaccines, as well as the incremental benefit of improved over standard vaccines on morbidity and mortality, can more clearly contextualise their relative benefits. The burden averted represents the additional influenza‐associated events (e.g., cases, medical attendances, hospitalisations and deaths) that would have been expected to occur in the absence of population vaccination [11, 13, 14]. Various approaches exist, but they commonly use available VE estimates, vaccination coverage and influenza‐associated events to estimate the number of events that would have been expected to occur in a counterfactual scenario without influenza vaccination in the population. Lewis et al. [12] extended this methodology to calculate the potential incremental benefits of hypothetical improved vaccines by applying nominated positive values of rVE to the absolute VE (aVE) of standard vaccines. The difference between the burden averted for improved and standard vaccination provided the theoretical incremental burden averted by improved vaccination [12].

Here, we aimed to estimate, for the population aged < 65 years in Australia, the additional morbidity and mortality that could be averted by use of vaccines with improved VE. Influenza vaccination VE could potentially be increased for this population group through policies that increase access to existing improved vaccines, or the development of improved vaccines based on new vaccine technologies.

## 2 Methods

This study estimates the number of influenza notifications, hospitalisations and deaths that would be expected to occur without a vaccine and would be avoided with current and hypothetically improved VE in a population aged < 65 years in Australia.

Data from 1 January 2015 to 31 December 2019 were included in the study. These years represent a range of different influenza seasons, in terms of severity, circulating subtypes and VE [15, 16], and occurred prior to the dramatic reduction of influenza transmission that occurred during the COVID‐19 pandemic [17]. All analyses were conducted using Microsoft Excel.

In Australia, vaccination is recommended for everyone aged ≥ 6 months [18]. However, the overall coverage rate was < 40% in 2022, with substantial variation by age, ranging from 70% in persons aged ≥ 65 years to 23% in 5‐ to 14‐year‐olds [19]. Under the Australian National Immunisation Program (NIP), vaccines are provided at no charge to groups at increased risk of severe influenza. In 2015, this included pregnant women, Aboriginal and/or Torres Strait Islanders aged < 5 years or ≥ 15 years, individuals aged ≥ 6 months with specified chronic medical conditions and those aged ≥ 65 years old [18]. Since the severe 2017 season [20], children aged 6 months to 5 years were included in many state‐funded programs in 2018 and in the NIP in 2019. Aboriginal and Torres Strait Islander individuals aged 5–15 years were included in the NIP in 2019. In addition, enhanced (adjuvanted or high‐dose) vaccines with improved effectiveness were included in the NIP for the ≥ 65‐year age group in 2018, with all other groups continuing to receive standard inactivated vaccines [18, 21].

Ethical approval was not required as only nonidentifiable aggregate data were used. The data from the National Hospital Morbidity Database were provided by the Australian Institute of Health and Welfare. All other data used were published estimates.

### 2.1 Data Sources

#### 2.1.1 VE and Coverage Estimates, 2015–2019

The primary analysis applied aVE estimates against laboratory confirmed influenza in general practice obtained from the published literature (Table 1) [22, 23, 24, 25, 26]. Seasonal influenza vaccine coverage for the study population were derived from annual estimates of age‐specific coverage (Table 1). Total coverage was estimated for the study population from age‐ and season‐specific coverage estimates according to weighted population size [27, 28, 29, 30, 31, 32, 33, 34, 35]. As in the Lewis et al.'s publication [12], in our analyses, we assumed the aVE and coverage point estimates were true, unbiased estimates.

**TABLE 1:** Published absolute influenza vaccine effectiveness and coverage in the Australian general population [22, 23, 24, 25, 26, 27, 28, 29, 30, 31, 32, 33].

Age group	2015	2016	2017	2018	2019
Absolute vaccine effectiveness					
All ages, primary care setting	54% [21]	40% [22]	33% [25]	68% [24]	48% [23]
Vaccine coverage					
6 months to < 5 years	3.0% [27]	3.0% [26]	5.6% [28]	26% [30]	43% [29]
5–17 years	5.0% [31]	5.0% [31]	5.0% [31]	5.0% [31]	20% [34]^a^
18–49 years	25% [32]	30%^b^	35%^b^	40%^b^	45% [33]
50–64 years^c^	46% [32, 33]	46% [32, 33]	46% [32, 33]	46% [32, 33]	46% [32, 33]

^a^ Coverage estimated as a weighted average of coverage by age group: 24.1% for 5–< 10 years; 18.8% for 10–< 15 years; 15.6% for 15–< 20 years.

^b^ Coverage assumed to increase progressively from 25% in 2015 [32] to 45% in 2019 [33].

^c^ Coverage estimates for 2014 and 2019 (46%) extended to all years [32, 33].

#### 2.1.2 Notifications

Laboratory‐confirmed influenza is a notifiable disease in Australia. Observed age and season‐specific notifications of laboratory confirmed influenza were obtained from the publicly available National Notifiable Diseases Surveillance System (NNDSS) influenza dataset [36].

#### 2.1.3 Hospitalisations

For 2015 through 2017, we used published modelled estimates of age‐specific influenza‐associated respiratory hospitalisations in Australia [3]. Population rates of respiratory admissions attributable to influenza were estimated by regressing weekly respiratory hospitalisations on a proxy measure of the relative weekly incidence of seasonal influenza infection [3]. Respiratory hospital admissions were defined by the presence of an *International Statistical Classification of Diseases and Related Health Problems, Tenth Revision, Australian Modification* (ICD‐10‐AM) respiratory diagnostic code, J00–J99, in the primary diagnosis field.

For 2018 and 2019, modelled estimates of excess influenza‐associated hospital admissions were unavailable. We therefore also used age‐specific hospitalisations for 2015 through 2019 from the Australian Institute of Health and Welfare's National Hospital Morbidity Database that had influenza recorded as a diagnosis (‘influenza‐coded hospitalisations’). Influenza was based on an ICD‐10‐AM code of J09 through J11. To maximise ascertainment of influenza‐associated admissions, records were selected if J09–J11 were recorded in any of the multiple available diagnostic fields [37]. Admissions with a care type of hospital boarder, posthumous organ donor or unqualified neonate were excluded. To avoid double counting, admissions in Western Australia with a status of ‘Inter‐hospital contracted patient to private sector hospital’ were excluded.

#### 2.1.4 Deaths

For influenza‐associated respiratory (J00–J99) and all‐cause excess deaths, we used published estimates that were derived from a regression method similar to that described for hospitalisations [2]. Estimates were used for the population aged < 65 years based on the availability of modelled estimates. Excess respiratory deaths were only available for 2015 through 2018, while estimated excess all‐cause deaths were available for 2015 through 2019 [2].

### 2.2 VE and Burden Averted

#### 2.2.1 aVE and Difference in aVE of Improved Vaccines

The aVE of the improved vaccine was estimated for the 2015–2019 seasons using the formula derived by Lewis et al. [12]:

$${\mathrm{aVE}}_{\text{Improved}}=\mathrm{rVE}\times \left(1-{\mathrm{aVE}}_{\text{Standard}}\right)+{\mathrm{aVE}}_{\text{Standard}}$$

where ${\mathrm{aVE}}_{\text{Improved}}$ is the aVE of the improved vaccine, rVE is the relative effectiveness of the improved vaccine and ${\mathrm{aVE}}_{\text{Standard}}$ is the published estimate of aVE for the overall population for the vaccine of a given season. The percentage point difference in aVE was then estimated as the percentage point difference between the aVE of the improved and standard vaccines:

$$\text{Difference in}\;\mathrm{aVE}={\mathrm{aVE}}_{\text{Improved}}-{\mathrm{aVE}}_{\text{Standard}}$$

All VE estimates were included as fractions in calculations but are presented as percentages.

The primary analyses were performed for rVE estimates of 5%, 15% and 30%. These align with published estimates of the relative efficacy/effectiveness of improved (cell‐based, recombinant, high‐dose and adjuvanted) vaccines compared to inactivated standard vaccines across a range of clinical outcomes, including medically attended influenza and influenza‐associated hospitalisation [38, 39, 40, 41, 42, 43].

#### 2.2.2 Estimating Burden Averted

The population burden of influenza in the scenario without vaccination was calculated for each infection outcome from the ‘baseline’, that is, observed or modelled frequencies under the prevailing vaccination scenario, using the following formula:

$${\text{Burden}}_{\text{Unvaccinated population}}=\frac{\text{Observed}\;\left(\text{or modelled}\right)\;\text{burden}}{\left(1-{\mathrm{aVE}}_{\text{Standard}}\times \text{Observed coverage}\right)}$$

where ${\mathrm{aVE}}_{\text{Standard}}$ and Observed coverage correspond to the published estimates presented in Table 1.

Following estimation of the influenza burden without influenza vaccination, we calculated the burden in the population in the scenario with vaccination, as

$${\text{Burden}}_{\text{Vaccinated}}={\text{Burden}}_{\text{Unvaccinated}}\times \left(1-\mathrm{aVE}\times \text{Assumed Coverage}\right)$$

where aVE corresponds to the standard or improved vaccine of interest. For simplicity, and noting increasing coverage trends in the younger population (Table 1), the study assumed coverage of 50% for both standard and improved vaccines.

The burden averted by each of the standard and improved vaccines was then determined as

$$\text{Burden averted}={\text{Burden}}_{\text{Unvaccinated}}-{\text{Burden}}_{\text{Vaccinated}}$$

And the incremental burden averted estimated as

$$\text{Incremental burden averted}={\text{Burden}}_{\text{Standard Vaccine}}-{\text{Burden}}_{\text{Improved Vaccine}}$$

#### 2.2.3 Sensitivity Analyses

For completeness, additional analyses were conducted using a wider range of hypothetical rVE estimates (5% increments from 5% through 40%) (Table S1). We also analysed the effect on the calculated burden averted of using estimates for alternative aVE estimated for hospital admissions based on data from the Influenza Complications Alert Network (FluCAN) (Tables S2, S3 and S5) [16, 20, 44, 45, 46]. Additionally, results are presented for the burden averted using alternative published regression estimates of influenza‐associated hospitalisation categories: acute respiratory infections (ARI; ICD10‐AM J00–J22 and J44.0), and pneumonia and influenza (P&I; ICD‐10‐AM J09–J18) (Tables S4 and S5) [3].

## 3 Results

Standard influenza VE estimates in primary care ranged from 33% in 2017 to 68% in 2018. In children aged 6 months to < 5 years, influenza vaccine coverage increased markedly from below 6% before 2018 to 43% by 2019, consistent with changes in eligibility for free vaccine in this age group during this period. From age 5 years, coverage increased with age to a maximum of 46% in 50‐ to 64‐year‐olds in all years. In 5‐ to 17‐year‐olds, coverage increased from a steady 5% in 2015–2018 to 20% in 2019, and in 18‐ to 49‐year‐olds, it increased from 25% in 2015 to 45% in 2019 (Table 1).

**FIGURE 1:**
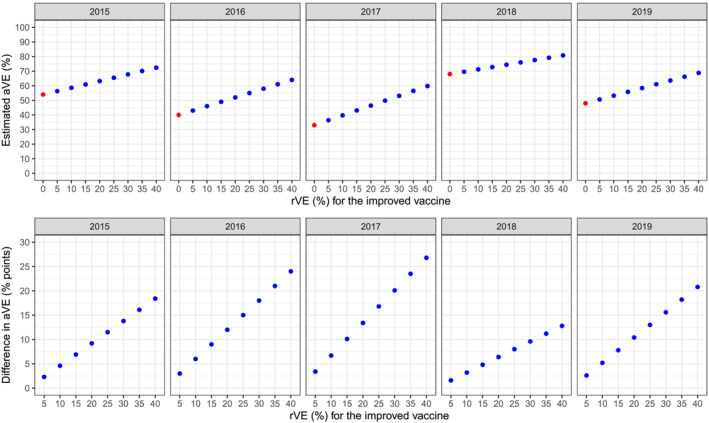
Estimated absolute vaccine effectiveness (aVE) and difference in aVE of an improved influenza vaccine under a range of relative vaccine effectiveness assumptions, Australia 2015–2019. The aVE for the standard vaccine, corresponding to rVE = 0, is shown in red. The estimated aVEs for improved influenza vaccines with rVEs ranging from 5% to 40% are show in blue. aVE = absolute vaccine effectiveness, rVE = relative vaccine effectiveness of the improved compared to the standard vaccine.

The estimated aVE of the improved vaccine increased with increasing aVE of the standard vaccine and increasing rVE. In 2017, the season with lowest standard vaccine aVE, the estimated aVE of the improved vaccine ranged from 36.4% to 59.8% for rVEs of 5% and 40%, respectively. In 2018, the season with the highest standard vaccine aVE, the corresponding improved vaccine aVE estimates were 69.6% and 80.8% (Figure 1). In contrast, the difference in aVE, expressed as the difference in percentage points, decreased as the standard aVE increased. In 2017, the difference in aVE ranged from 3.4% to 26.8% for rVEs of 5% and 40%, respectively, while for 2018, the corresponding difference in aVE ranged from 1.6% to 12.8% (Figure 1).

The burden of influenza infection outcomes under the baseline scenario in the population aged < 65 years in Australia was highest in 2017 and 2019 and generally lowest in 2018. There were 265,104 influenza notifications in 2019, 196,510 in 2017 and 48,970 in 2018. There were 26,939 influenza‐coded hospitalisations in 2019, 21,919 in 2017 and 8994 in 2018. Modelled influenza‐associated respiratory hospitalisations for 2015–2017 were similar to influenza‐coded hospitalisations. However, influenza‐associated deaths were highest in 2017 for both respiratory (223) and all‐cause deaths (527), followed by 2019, for which only all‐cause deaths' data were available (459). The lowest all‐cause death estimate was 254 in 2016 (Table 2).

**TABLE 2:** Burden of influenza‐associated events for baseline standard vaccine coverage and estimated for no vaccination and 50% coverage in the population aged < 65 years, Australia, 2015–2019.

Influenza‐associated events	2015	2016	2017	2018	2019
Influenza notifications^a^					
Standard vaccine, baseline coverage	84,849	69,759	196,510	48,970	265,104
No vaccination	95,490	77,669	215,340	63,219	324,134
Standard vaccine, 50% coverage	69,708	62,135	179,809	41,724	246,342
Influenza‐coded hospitalisations^a^					
Standard vaccine, baseline coverage	10,367	10,065	21,919	8994	26,939
No vaccination	11,911	11,356	24,408	11,950	33,627
Standard vaccine, 50% coverage	8695	9085	20,381	7887	25,557
Influenza‐associated respiratory hospitalisations^b^					
Standard vaccine, baseline coverage	11,211	9964	18,823	NA	NA
No vaccination	13,316	11,381	21,153	NA	NA
Standard vaccine, 50% coverage	9721	9105	17,663	NA	NA
Influenza‐associated respiratory deaths^b^					
Standard vaccine, baseline coverage	64	106	223	77	NA
No vaccination	74	119	247	100	NA
Standard vaccine, 50% coverage	54	95	206	66	NA
Influenza‐associated all‐cause deaths^b^					
Standard vaccine, baseline coverage	306	254	587	303	456
No vaccination	352	284	650	393	565
Standard vaccine, 50% coverage	257	227	543	259	430

^a^ Estimates based on observed data: NNDSS influenza notifications [35] and hospital admissions recorded in the Australian Institute of Health and Welfare National Hospital Morbidity Database, with a J09–J11 code in any diagnostic field.

^b^ Estimates based on modelled influenza‐associated hospitalisations [3] and deaths [2].

Over the study period, the standard influenza vaccine with 50% coverage was estimated to avert an annual average of 35,227 notifications, 4330 influenza‐coded hospitalisations, 3120 influenza‐associated respiratory hospitalisations, 30 influenza‐associated respiratory deaths and 106 influenza‐associated all cause deaths. There was considerable variation among seasons, with 15,534 notifications, 2271 influenza‐coded hospitalisations and 57 all‐cause influenza‐associated deaths averted in 2016, compared to 77,792, 8071 and 137 in 2019, respectively. The magnitude of the burden averted is a function of both the season‐specific severity and the aVE of the influenza vaccine (Table 3).

**TABLE 3:** Estimated burden of influenza‐associated events averted by standard and improved vaccines, assuming 50% coverage in the population aged < 65 years, Australia, 2015–2019.

Influenza‐associated events	rVE^a^	2015	2016	2017	2018	2019
Influenza notifications^a^						
Averted by standard vaccine	0%	25,782	15,534	35,531	21,494	77,792
Incremental burden averted by improved vaccine	5%	1098	1165	3607	506	4214
	15%	3294	3495	10,821	1517	12,641
	30%	6589	6990	21,642	3035	25,282
Influenza‐coded hospitalisations^b^						
Averted by standard vaccine	0%	3216	2271	4027	4063	8071
Incremental burden averted by improved vaccine	5%	137	170	409	96	437
	15%	411	511	1227	287	1311
	30%	822	1022	2453	574	2623
Influenza‐associated respiratory hospitalisations^c^						
Averted by standard vaccine	0%	3595	2276	3490	NA	NA
Incremental burden averted by improved vaccine	5%	153	171	354	NA	NA
	15%	459	512	1063	NA	NA
	30%	919	1024	2126	NA	NA
Influenza‐associated respiratory deaths^c^						
Averted by standard vaccine	0%	20	24	41	34	NA
Incremental burden averted by improved vaccine	5%	1	2	4	1	NA
	15%	3	5	12	2	NA
	30%	5	11	25	5	NA
Influenza‐associated all‐cause deaths^c^						
Averted by standard vaccine	0%	95	57	107	134	136
Incremental burden averted by improved vaccine	5%	4	4	11	3	7
	15%	12	13	33	9	22
	30%	24	26	65	19	44

^a^ rVE = relative vaccine effectiveness of the improved compared to the standard vaccine.

^b^ Estimates based on observed data: NNDSS influenza notifications [35] and hospital admissions recorded in the Australian Institute of Health and Welfare National Hospital Morbidity Database, with a J09–J11 code in any diagnostic field.

^c^ Estimates based on modelled influenza‐associated hospitalisations [3] and deaths [2].

A hypothetical improved vaccine was estimated to have had the highest additional benefit in 2019 or 2017 and the lowest impact in 2018 (Table 3). Assuming an rVE of 15% with 50% coverage, the improved vaccine would have incrementally averted 1517 to 12,641 influenza notifications, 287 to 1311 influenza‐coded hospitalisations and 9 to 33 modelled all‐cause influenza deaths per year compared to the standard vaccine (Table 3). Increasing the rVE to 30% would double those estimates. For a lower rVE of 5%, an improved vaccine with 50% coverage was estimated to incrementally avert 506 to 4214 influenza notifications, 96 to 437 influenza‐coded hospitalisations and 3 to 11 all‐cause influenza‐associated deaths (Table 3).

The results of sensitivity analyses are presented in Tables S1–S5. Similar trends were observed, with the highest burden averted estimated for the 2017 and 2019 seasons.

## 4 Discussion

We have provided counterfactual estimates of the burden of influenza in an Australian population aged < 65 years without influenza vaccination and the potential burden averted by standard and improved vaccines. The difference in aVE of an improved vaccine is dependent on both its rVE and the aVE of the standard vaccine. Higher rVE translates to higher aVE and difference in aVE of the improved vaccine. However, the difference in aVE of the improved vaccine is inversely proportional to the aVE of the standard vaccine [12]. Thus, for the Australian examples presented, the estimated difference in aVE of an improved vaccine is highest in 2017 and lowest in 2018, the seasons with the lowest (33%) and highest (68%) observed standard vaccine aVE, respectively.

The magnitude of the incremental burden averted by an improved vaccine is a function of both the season‐specific severity and the difference in aVE of the vaccine [12]. Thus, for Australia, the highest additional burden averted was estimated for seasons with higher severity, such as 2019, or lower aVE of the standard vaccine, such as 2017. Assuming 50% coverage between 2015 and 2019, an improved vaccine with 15% rVE was estimated to incrementally avert 1517 to 12,641 influenza notifications, 287 to 1311 influenza‐coded hospitalisations and 9 to 33 modelled all‐cause influenza deaths per year.

Our study used both modelled (influenza‐associated) and observed (influenza‐coded) hospitalisation estimates. Reliance on diagnosis codes only can underestimate influenza‐associated hospitalisations [2, 3]. This can occur because not all patients are tested for influenza and some positive test results are not recorded [2, 37]. Under‐recognition of influenza as a causative or exacerbating factor for respiratory and nonrespiratory admissions has been well described [47, 48]. In contrast to previous studies, we found a notable similarity between directly ascertained influenza‐coded hospitalisations and modelled influenza‐associated respiratory hospitalisations. The similarity may reflect our inclusion of influenza diagnoses in any position in the diagnostic record or increased use of diagnostics in the hospital setting. Indeed, Moore et al. found that their sensitivity to identify admitted individuals positive for influenza was reduced from 86% to 74% when only the primary diagnostic field was analysed, compared to the first 20 diagnoses [37]. In a more recent study conducted in 2016–2017, Wabe et al. determined influenza‐coded ICD codes to be 86% sensitive for primary or secondary diagnosis, and this increased to 93% when test results were available prior to discharge [49]. It may be that as the accessibility, frequency and rapidity of testing have increased over time, ICD codes are now able to provide more sensitive estimates of influenza‐associated hospitalisations. On the other hand, including more diagnosis fields increases the risk of admissions with incidental infection being included [3].

Our study has limitations. The results depend on the accuracy of the outcomes and aVE estimates we used. We used estimates of aVE for the whole Australian population. These are likely an underestimate of VE in the < 65 year population as lower VE has been observed for influenza vaccination in populations aged ≥ 65 years [10]. Notifications underestimate infections since they only include infections for which health care was sought, a test conducted, a diagnosis made and notification to health authorities completed. Nevertheless, notifications provide a useful indication of known infections. The regression‐based estimates of influenza‐associated morbidity and mortality have been widely used and are accepted as providing the best approximation of influenza‐associated outcomes [1], although methods can produce wide variation in estimates. The similarity between observed influenza‐coded and modelled hospitalisation and the burden averted estimates increases our confidence in the results.

For simplicity, we did not account for the uncertainty in aVE estimates, which can have wide confidence intervals, or uncertainty in other outcomes. Nevertheless, the sensitivity analyses of alternative aVE estimates from the hospital setting and alternative modelled estimates of estimated influenza‐associated hospitalisations and deaths demonstrated similar trends. We assumed 50% vaccination coverage for both standard and improved vaccines. Although somewhat higher than current levels in the Australian community, it is a plausible continuation of the trend of increasing influenza vaccination rates in Australia observed among younger age groups. The theoretical values of rVE used were not intended to reflect a specific vaccine. We have also assumed that the same aVE and rVE applies to all infection outcomes, which may not be accurate.

We did not consider the indirect population effects of vaccination through decreased population transmission or herd immunity. Indirect benefits increase with VE [50] and higher levels of coverage, particularly in the paediatric population [51, 52], and can exceed direct benefits [53]. Influenza circulation is associated with increased cardiovascular and all‐cause hospitalisations [48]. Incorporating these factors requires more sophisticate modelling approaches.

## 5 Conclusion

Standard influenza vaccination achieves substantial population health benefits in terms of averted notifications, hospitalisations and deaths among the Australian population aged < 65 years. We have shown that introduction of an improved vaccine with plausible rVE can have substantial additional impact in terms of the hospital and mortality burden averted, particularly in seasons with low standard VE and high influenza activity. As in other settings, this quantification is useful to contextualise the complex interpretation of rVE estimates across different seasons and inform policies designed to increase access and uptake of improved vaccines in the < 65 year age group [11, 13, 14].

## Author Contributions

**Alicia N. Stein:** Conceptualization; Data curation; Formal analysis; Investigation; Methodology; Project administration; Writing – original draft; Writing – review and editing. **Catherine G. A. Pendrey:** Conceptualization; Formal analysis; Investigation; Methodology; Validation; Writing – original draft; Writing – review and editing. **David J. Muscatello:** Conceptualization; Formal analysis; Investigation; Methodology; Validation; Writing – original draft; Writing – review and editing. **Paul G. Van Buynder:** Conceptualization; Investigation; Writing – review and editing. **James E. Fielding:** Conceptualization; Investigation; Writing – review and editing. **Jason C. Menche:** Conceptualization; Investigation; Project administration; Writing – review and editing. **Sheena G. Sullivan:** Conceptualization; Investigation; Methodology; Supervision; Writing – original draft; Writing – review and editing.

## Ethics Statement

Ethical approval was not required as only nonidentifiable aggregate data were used.

## Conflicts of Interest

ANS and JCM are employees of CSL Seqirus and own shares in CSL Pty Ltd; DJM has had unpaid participation in an influenza advisory board meeting for CSL Seqirus, with travel and meals provided. SGS reports receipt of consulting fees for CSL Seqirus, Pfizer, Moderna and EvoHealth; PGVB is a member of the Australian Influenza Vaccine Committee; CGAP and JEF have nothing to declare.

## Supporting information


**Table S1.** Estimates for influenza notifications, hospitalisations and deaths and burden averted assuming 50% coverage of standard or improved influenza vaccines in the population aged < 65 years, Australia, 2015–2019.
**Table S2.** Estimated absolute vaccine effectiveness (aVE) and difference in aVE of an improved influenza vaccine under a range of relative vaccine effectiveness assumptions using FluCAN estimates of standard influenza vaccine effectiveness [4, 5, 6, 7, 8, 9].
**Table S3.** Estimates for influenza notifications, hospitalisations and deaths and burden averted assuming 50% coverage of standard or improved influenza vaccines in the population aged < 65 years using FluCAN estimates of standard influenza vaccine effectiveness.
**Table S4.** Estimates for influenza‐associated acute respiratory infection hospitalisations and pneumonia & influenza hospitalisations and deaths and burden averted assuming 50% coverage of standard or improved influenza vaccines in the population aged < 65 years, Australia, 2015–2018.
**Table S5.** Estimates of burden of influenza‐associated acute respiratory infection hospitalisations and pneumonia & influenza hospitalisations and deaths, and burden averted by standard or improved influenza vaccines assuming 50% coverage in the population aged < 65 years using FluCAN absolute vaccine effectiveness, Australia, 2015–2018.

## Data Availability

Observed age and season‐specific notifications of laboratory confirmed influenza were obtained from the National Notifiable Diseases Surveillance System (NNDSS) influenza dataset, available at National Notifiable Diseases Surveillance System (NNDSS) public datasets | Australian Government Department of Health and Aged Care. Hospitalisation data were from the National Hospital Morbidity Database provided by the Australian Institute of Health and Welfare and are available on request from http://www.aihw.gov.au/.
