# Supplementary material for: Estimates of Seasonal Influenza Burden That Could Be Averted by Improved Influenza Vaccines in the Australian Population Aged Under 65 Years, 2015–2019
Source: Influenza Other Respir Viruses. 2024 Apr 18;18(4):e13289. doi: 10.1111/irv.13289 (PMC11026859; doi:10.1111/irv.13289)
Supplement: Supplementary file 1 — Table S1. Estimates for influenza notifications, hospitalisations and deaths and burden averted assuming 50% coverage of standard or improved influenza vaccines in the population aged < 65 years, Australia, 2015–2019. Table S2. Estimated absolute vaccine effectiveness (aVE) and difference in aVE of an improved influenza vaccine under a range of relative vaccine effectiveness assumptions using FluCAN estimates of standard influenza vaccine effectiveness [4, 5, 6, 7, 8, 9]. Table S3. Estimates for influenza notifications, hospitalisations and deaths and burden averted assuming 50% coverage of standard or improved influenza vaccines in the population aged < 65 years using FluCAN estimates of standard influenza vaccine effectiveness. Table S4. Estimates for influenza‐associated acute respiratory infection hospitalisations and pneumonia & influenza hospitalisations and deaths and burden averted assuming 50% coverage of standard or improved influenza vaccines in the population aged < 65 years, Australia, 2015–2018. Table S5. Estimates of burden of influenza‐associated acute respiratory infection hospitalisations and pneumonia & influenza hospitalisations and deaths, and burden averted by standard or improved influenza vaccines assuming 50% coverage in the population aged < 65 years using FluCAN absolute vaccine effectiveness, Australia, 2015–2018. [file IRV-18-e13289-s001.docx]

## Supplementary Material

Supplementary Table 1. Estimates for influenza notifications, hospitalisations and deaths and burden averted assuming 50% coverage of standard or improved influenza vaccines in the population aged < 65 years, Australia, 2015-2019

| Influenza-associated events | Coverage | rVE ^a^ | 2015 | 2016 | 2017 | 2018 | 2019 |
| --- | --- | --- | --- | --- | --- | --- | --- |
| **Influenza notifications ^b^** | | | | | | | |
| Standard vaccine, baseline | As per Table 1 |  | 84,849 | 69,759 | 196,510 | 48,970 | 265,104 |
| No vaccination | 0% |  | 95,490 | 77,669 | 215,340 | 63,219 | 324,134 |
| Standard vaccine | 50% |  | 69,708 | 62,135 | 179,809 | 41,724 | 246,342 |
| Averted by standard vaccine | 50% |  | 25,782 | 15,534 | 35,531 | 21,494 | 77,792 |
| Incremental burden averted by improved vaccine | 50% | 5% | 1,098 | 1,165 | 3,607 | 506 | 4,214 |
|  |  | 10% | 2,196 | 2,330 | 7,214 | 1,012 | 8,427 |
|  |  | 15% | 3,294 | 3,495 | 10,821 | 1,517 | 12,641 |
|  |  | 20% | 4,393 | 4,660 | 14,428 | 2,023 | 16,855 |
|  |  | 25% | 5,491 | 5,825 | 18,035 | 2,529 | 21,069 |
|  |  | 30% | 6,589 | 6,990 | 21,642 | 3,035 | 25,282 |
|  |  | 35% | 7,687 | 8,155 | 25,249 | 3,540 | 29,496 |
|  |  | 40% | 8,785 | 9,320 | 28,856 | 4,046 | 33,710 |
| **Influenza-coded hospitalisations ^b^** | | | | | | | |
| Standard vaccine, baseline | As per Table 1 |  | 10,367 | 10,065 | 21,919 | 8,994 | 26,939 |
| No vaccination | 0% |  | 11,911 | 11,356 | 24,408 | 11,950 | 33,627 |
| Standard vaccine | 50% |  | 8,695 | 9,085 | 20,381 | 7,887 | 25,557 |
| Averted by standard vaccine | 50% |  | 3,216 | 2,271 | 4,027 | 4,063 | 8,071 |
| Incremental burden averted by improved vaccine | 50% | 5% | 137 | 170 | 409 | 96 | 437 |
|  |  | 10% | 274 | 341 | 818 | 191 | 874 |
|  |  | 15% | 411 | 511 | 1,227 | 287 | 1,311 |
|  |  | 20% | 548 | 681 | 1,635 | 382 | 1,749 |
|  |  | 25% | 685 | 852 | 2,044 | 478 | 2,186 |
|  |  | 30% | 822 | 1,022 | 2,453 | 574 | 2,623 |
|  |  | 35% | 959 | 1,192 | 2,862 | 669 | 3,060 |
|  |  | 40% | 1,096 | 1,363 | 3,271 | 765 | 3,497 |
| **Influenza-associated respiratory hospitalisations ^c^** | | | | | | | |
| Standard vaccine, baseline | As per Table 1 |  | 11,211 | 9,964 | 18,823 | NA | NA |
| No vaccination | 0% |  | 13,316 | 11,381 | 21,153 | NA | NA |
| Standard vaccine | 50% |  | 9,721 | 9,105 | 17,663 | NA | NA |
| Averted by standard vaccine | 50% |  | 3,595 | 2,276 | 3,490 | NA | NA |
| Incremental burden averted by improved vaccine | 50% | 5% | 153 | 171 | 354 | NA | NA |
|  |  | 10% | 306 | 341 | 709 | NA | NA |
|  |  | 15% | 459 | 512 | 1,063 | NA | NA |
|  |  | 20% | 613 | 683 | 1,417 | NA | NA |
|  |  | 25% | 766 | 854 | 1,772 | NA | NA |
|  |  | 30% | 919 | 1,024 | 2,126 | NA | NA |
|  |  | 35% | 1,072 | 1,195 | 2,480 | NA | NA |
|  |  | 40% | 1,225 | 1,366 | 2,835 | NA | NA |
| **Influenza-associated respiratory deaths ^c^** | | | | | | | |
| Standard vaccine, baseline | As per Table 1 |  | 64 | 106 | 223 | 77 | NA |
| No vaccination | 0% |  | 74 | 119 | 247 | 100 | NA |
| Standard vaccine | 50% |  | 54 | 95 | 206 | 66 | NA |
| Averted by standard vaccine | 50% |  | 20 | 24 | 41 | 34 | NA |
| Incremental burden averted by improved vaccine | 50% | 5% | 0.8 | 1.8 | 4.1 | 0.8 | NA |
|  |  | 10% | 1.7 | 3.6 | 8.3 | 1.6 | NA |
|  |  | 15% | 2.5 | 5.3 | 12.4 | 2.4 | NA |
|  |  | 20% | 3.4 | 7.1 | 16.5 | 3.2 | NA |
|  |  | 25% | 4.2 | 8.9 | 20.7 | 4.0 | NA |
|  |  | 30% | 5.1 | 10.7 | 24.8 | 4.8 | NA |
|  |  | 35% | 5.9 | 12.5 | 29.0 | 5.6 | NA |
|  |  | 40% | 6.8 | 14.2 | 33.1 | 6.4 | NA |
| **Influenza-associated all-cause deaths ^c^** | | | | | | | |
| Standard vaccine, baseline | As per Table 1 |  | 306 | 254 | 587 | 303 | 456 |
| No vaccination | 0% |  | 352 | 284 | 650 | 393 | 565 |
| Standard vaccine | 50% |  | 257 | 227 | 543 | 259 | 430 |
| Averted by standard vaccine | 50% |  | 95 | 57 | 107 | 134 | 136 |
| Incremental burden averted by improved vaccine | 50% | 5% | 4.0 | 4.3 | 10.9 | 3.1 | 7.3 |
|  |  | 10% | 8.1 | 8.5 | 21.8 | 6.3 | 14.7 |
|  |  | 15% | 12.1 | 12.8 | 32.7 | 9.4 | 22.0 |
|  |  | 20% | 16.2 | 17.1 | 43.6 | 12.6 | 29.4 |
|  |  | 25% | 20.2 | 21.3 | 54.4 | 15.7 | 36.7 |
|  |  | 30% | 24.3 | 25.6 | 65.3 | 18.8 | 44.1 |
|  |  | 35% | 28.3 | 29.8 | 76.2 | 22.0 | 51.4 |
|  |  | 40% | 32.3 | 34.1 | 87.1 | 25.1 | 58.8 |

^a^ rVE indicates the relative vaccine effectiveness of the improved compared to the standard vaccine.

^b^ Estimates based on observed data: NNDSS influenza notifications^1^ and hospital admissions recorded in the Australian Institute of Health and Welfare National Hospital Morbidity Database, with a J09-J11 code in any diagnostic field.

^c^ Estimates based on modelled influenza-associated hospitalisations^3^ and deaths.^2^

Supplementary Table 2. Estimated absolute vaccine effectiveness (aVE) and difference in aVE of an improved influenza vaccine under a range of relative vaccine effectiveness assumptions using FluCAN estimates of standard influenza vaccine effectiveness^4-9^

| Season | aVE Standard | rVE for the improved vaccine | | | | | | | |
| --- | --- | --- | --- | --- | --- | --- | --- | --- | --- |
|  |  | 5% | 10% | 15% | 20% | 25% | 30% | 35% | 40% |
|  |  | Estimated aVE for the improved vaccine with specified rVE | | | | | | | |
| 2015 | 45.0%^4^ | 47.8% | 50.5% | 53.3% | 56.0% | 58.8% | 61.5% | 64.3% | 67.0% |
| 2016 | 13.0%^5^ | 17.4% | 21.7% | 26.1% | 30.4% | 34.8% | 39.1% | 43.5% | 47.8% |
| 2017 | 23.0%^6^ | 26.9% | 30.7% | 34.6% | 38.4% | 42.3% | 46.1% | 50.0% | 53.8% |
| 2018 | 52.0%^9^ | 54.4% | 56.8% | 59.2% | 61.6% | 64.0% | 66.4% | 68.8% | 71.2% |
| 2019 | 52.0%^8^ | 54.4% | 56.8% | 59.2% | 61.6% | 64.0% | 66.4% | 68.8% | 71.2% |
|  |  | Difference in aVE (percentage point difference) for specified rVE | | | | | | | |
| 2015 | 45.0%^4^ | 2.8% | 5.5% | 8.3% | 11.0% | 13.8% | 16.5% | 19.3% | 22.0% |
| 2016 | 13.0%^5^ | 4.4% | 8.7% | 13.1% | 17.4% | 21.8% | 26.1% | 30.5% | 34.8% |
| 2017 | 23.0%^6^ | 3.9% | 7.7% | 11.6% | 15.4% | 19.3% | 23.1% | 27.0% | 30.8% |
| 2018 | 52.0%^9^ | 2.4% | 4.8% | 7.2% | 9.6% | 12.0% | 14.4% | 16.8% | 19.2% |
| 2019 | 52.0%^8^ | 2.4% | 4.8% | 7.2% | 9.6% | 12.0% | 14.4% | 16.8% | 19.2% |

aVE = Absolute vaccine effectiveness; rVE = Relative vaccine effectiveness of the improved compared to the standard vaccine.

Supplementary Table 3. Estimates for influenza notifications, hospitalisations and deaths and burden averted assuming 50% coverage of standard or improved influenza vaccines in the population aged < 65 years using FluCAN estimates of standard influenza vaccine effectiveness

| Influenza-associated events | Coverage | rVE ^a^ | 2015 | 2016 | 2017 | 2018 | 2019 |
| --- | --- | --- | --- | --- | --- | --- | --- |
| **Influenza notifications^a^** | | | | | | | |
| Standard vaccine, Baseline | As per Table 1 |  | 84,849 | 69,759 | 196,510 | 48,970 | 265,104 |
| No vaccination | 0% |  | 95,490 | 77,669 | 215,340 | 63,219 | 324,134 |
| Standard vaccine | 50% |  | 74,005 | 72,621 | 190,576 | 46,782 | 239,859 |
| Averted by standard vaccine | 50% |  | 21,485 | 5,049 | 24,764 | 16,437 | 84,275 |
| Incremental burden averted by improved vaccine | 50% | 5% | 1,313 | 1,689 | 4,145 | 759 | 3,890 |
|  |  | 10% | 2,626 | 3,379 | 8,291 | 1,517 | 7,779 |
|  |  | 15% | 3,939 | 5,068 | 12,436 | 2,276 | 11,669 |
|  |  | 20% | 5,252 | 6,757 | 16,581 | 3,035 | 15,558 |
|  |  | 25% | 6,565 | 8,447 | 20,726 | 3,793 | 19,448 |
|  |  | 30% | 7,878 | 10,136 | 24,872 | 4,552 | 23,338 |
|  |  | 35% | 9,191 | 11,825 | 29,017 | 5,310 | 27,227 |
|  |  | 40% | 10,504 | 13,514 | 33,162 | 6,069 | 31,117 |
| **Influenza-coded hospitalisations ^b^** | | | | | | | |
| Standard vaccine, Baseline | As per Table 1 |  | 10,367 | 10,065 | 21,919 | 8,994 | 26,939 |
| No vaccination | 0% |  | 11,604 | 10,964 | 24,030 | 11,467 | 34,109 |
| Standard vaccine | 50% |  | 8,993 | 10,251 | 21,267 | 8,486 | 25,240 |
| Averted by standard vaccine | 50% |  | 2,611 | 713 | 2,763 | 2,981 | 8,868 |
| Incremental burden averted by improved vaccine | 50% | 5% | 160 | 238 | 463 | 138 | 409 |
|  |  | 10% | 319 | 477 | 9255 | 275 | 819 |
|  |  | 15% | 479 | 715 | 1,388 | 413 | 1,228 |
|  |  | 20% | 638 | 954 | 1,850 | 550 | 1,637 |
|  |  | 25% | 798 | 1,192 | 2,313 | 688 | 2,047 |
|  |  | 30% | 957 | 1,431 | 2,775 | 826 | 2,456 |
|  |  | 35% | 1,117 | 1,669 | 3,238 | 963 | 2,865 |
|  |  | 40% | 1,276 | 1,908 | 3,701 | 1,101 | 3,274 |
| **Influenza-associated respiratory hospitalisations ^c^** | | | | | | | |
| Standard vaccine, Baseline | As per Table 1 |  | 11,211 | 9,964 | 18,823 | NA | NA |
| No vaccination | 0% |  | 12,898 | 10,376 | 20,378 | NA | NA |
| Standard vaccine | 50% |  | 9,996 | 9,702 | 18,035 | NA | NA |
| Averted by standard vaccine | 50% |  | 2,902 | 674 | 2,343 | NA | NA |
| Incremental burden averted by improved vaccine | 50% | 5% | 177 | 226 | 392 | NA | NA |
|  |  | 10% | 355 | 451 | 785 | NA | NA |
|  |  | 15% | 532 | 677 | 1,177 | NA | NA |
|  |  | 20% | 709 | 903 | 1,569 | NA | NA |
|  |  | 25% | 887 | 1,128 | 1,961 | NA | NA |
|  |  | 30% | 1,064 | 1,354 | 2,354 | NA | NA |
|  |  | 35% | 1,241 | 1,580 | 2,746 | NA | NA |
|  |  | 40% | 1,419 | 1,805 | 3,138 | NA | NA |
| **Influenza-associated respiratory deaths ^c^** | | | | | | | |
| Standard vaccine, Baseline | As per Table 1 |  | 64 | 106 | 223 | 77 | NA |
| No vaccination | 0% |  | 72 | 110 | 239 | 93 | NA |
| Standard vaccine | 50% |  | 56 | 103 | 212 | 69 | NA |
| Averted by standard vaccine | 50% |  | 16 | 7 | 27 | 24 | NA |
| Incremental burden averted by improved vaccine | 50% | 5% | 1.0 | 2.4 | 4.6 | 1.1 | NA |
|  |  | 10% | 2.0 | 4.8 | 9.2 | 2.2 | NA |
|  |  | 15% | 3.0 | 7.2 | 13.8 | 3.4 | NA |
|  |  | 20% | 3.9 | 9.6 | 18.4 | 4.5 | NA |
|  |  | 25% | 4.9 | 11.9 | 23.0 | 5.6 | NA |
|  |  | 30% | 5.9 | 14.3 | 27.6 | 6.7 | NA |
|  |  | 35% | 6.9 | 16.7 | 32.2 | 7.8 | NA |
|  |  | 40% | 7.9 | 19.1 | 36.8 | 9.0 | NA |
| **Influenza-associated all-cause deaths ^c^** | | | | | | | |
| Standard vaccine, Baseline | As per Table 1 |  | 306 | 254 | 587 | 303 | 456 |
| No vaccination | 0% |  | 343 | 263 | 630 | 367 | 565 |
| Standard vaccine | 50% |  | 266 | 246 | 557 | 272 | 418 |
| Averted by standard vaccine | 50% |  | 77 | 17 | 73 | 95 | 147 |
| Incremental burden averted by improved vaccine | 50% | 5% | 4.7 | 5.7 | 12.1 | 4.4 | 6.8 |
|  |  | 10% | 9.4 | 11.4 | 24.2 | 8.8 | 13.6 |
|  |  | 15% | 14.1 | 17.2 | 36.4 | 13.2 | 20.3 |
|  |  | 20% | 18.9 | 22.9 | 48.5 | 17.6 | 27.1 |
|  |  | 25% | 23.6 | 28.6 | 60.6 | 22.0 | 33.9 |
|  |  | 30% | 28.3 | 34.3 | 72.7 | 26.4 | 40.7 |
|  |  | 35% | 33.0 | 40.1 | 84.8 | 30.8 | 47.5 |
|  |  | 40% | 37.7 | 45.8 | 97.0 | 35.2 | 54.3 |

^a^ rVE indicates the relative vaccine effectiveness of the improved compared to the standard vaccine.

^b^ Estimates based on observed data: NNDSS influenza notifications^1^ and hospital admissions recorded in the Australian Institute of Health and Welfare National Hospital Morbidity Database, with a J09-J11 code in any diagnostic field.

^c^ Estimates based on modelled influenza-associated hospitalisations^2^ and deaths^3^.

Supplementary Table 4. Estimates for influenza-associated acute respiratory infection hospitalisations and pneumonia & influenza hospitalisations and deaths and burden averted assuming 50% coverage of standard or improved influenza vaccines in the population aged < 65 years, Australia, 2015-2018

| Influenza-associated events | Coverage | rVE^a^ | 2015 | 2016 | 2017 | 2018 |
| --- | --- | --- | --- | --- | --- | --- |
| **Influenza-associated acute respiratory infection hospitalisations^b^** | | | | | | |
| Standard vaccine, Baseline | As per Table 1 |  | 11,540 | 10,255 | 19,363 | NA |
| No vaccination | 0% |  | 13,549 | 16,071 | 29,598 | NA |
| Standard vaccine | 50% |  | 9,825 | 12,857 | 24,714 | NA |
| Averted by standard vaccine | 50% |  | 3,634 | 3,214 | 4,884 | NA |
| Incremental burden averted by improved vaccine | 50% | 5% | 155 | 173 | 360 | NA |
|  |  | 10% | 310 | 346 | 720 | NA |
|  |  | 15% | 464 | 520 | 1,081 | NA |
|  |  | 20% | 619 | 693 | 1,441 | NA |
|  |  | 25% | 774 | 866 | 1,801 | NA |
|  |  | 30% | 929 | 1,039 | 2,161 | NA |
|  |  | 35% | 1,083 | 1,213 | 2,521 | NA |
|  |  | 40% | 1,238 | 1,386 | 2,882 | NA |
| **Influenza-associated pneumonia and influenza hospitalisations^b^** | | | | | | |
| Standard vaccine, Baseline | As per Table 1 |  | 8,768 | 7,789 | 14,700 | NA |
| No vaccination | 0% |  | 10,132 | 11,392 | 21,040 | NA |
| Standard vaccine | 50% |  | 7,396 | 9,113 | 17,568 | NA |
| Averted by standard vaccine | 50% |  | 2,736 | 2,278 | 3,472 | NA |
| Incremental burden averted by improved vaccine | 50% | 5% | 117 | 131 | 272 | NA |
|  |  | 10% | 233 | 261 | 544 | NA |
|  |  | 15% | 350 | 392 | 817 | NA |
|  |  | 20% | 466 | 523 | 1,089 | NA |
|  |  | 25% | 583 | 654 | 1,361 | NA |
|  |  | 30% | 699 | 784 | 1,633 | NA |
|  |  | 35% | 816 | 915 | 1,905 | NA |
|  |  | 40% | 932 | 1,046 | 2,178 | NA |
| **Influenza-associated pneumonia and influenza deaths^b^** | | | | | | |
| Standard vaccine, Baseline | As per Table 1 |  | 37 | 51 | 156 | 32 |
| No vaccination | 0% |  | 43 | 57 | 173 | 41 |
| Standard vaccine | 50% |  | 31 | 46 | 144 | 27 |
| Averted by standard vaccine | 50% |  | 12 | 11 | 29 | 14 |
| Incremental burden averted by improved vaccine | 50% | 5% | 0.5 | 0.9 | 2.9 | 0.3 |
|  |  | 10% | 1.0 | 1.7 | 5.8 | 0.7 |
|  |  | 15% | 1.5 | 2.6 | 8.7 | 1.0 |
|  |  | 20% | 2.0 | 3.4 | 11.6 | 1.3 |
|  |  | 25% | 2.4 | 4.3 | 14.5 | 1.7 |
|  |  | 30% | 2.9 | 5.1 | 17.4 | 2.0 |
|  |  | 35% | 3.4 | 6.0 | 20.3 | 2.3 |
|  |  | 40% | 3.9 | 6.8 | 23.1 | 2.7 |

^a^ rVE indicates the relative vaccine effectiveness of the improved compared to the standard vaccine.

^b^ Estimates based on modelled influenza-associated hospitalisations^2^ and deaths^3^. Data not available for 2019.

Supplementary Table 5. Estimates of burden of influenza-associated acute respiratory infection hospitalisations and pneumonia & influenza hospitalisations and deaths, and burden averted by standard or improved influenza vaccines assuming 50% coverage in the population aged < 65 years using FluCAN absolute vaccine effectiveness, Australia, 2015-2018

| Influenza-associated events | Coverage | rVE^a^ | 2015 | 2016 | 2017 | 2018 |
| --- | --- | --- | --- | --- | --- | --- |
| **Influenza-associated ARI hospitalisations^b^** | | | | | | |
| Standard vaccine, Baseline | As per Table 1 |  | 11,540 | 10,255 | 19,363 | NA |
| No vaccination | 0% |  | 13,079 | 10,632 | 20,794 | NA |
| Standard vaccine | 50% |  | 10,136 | 9,941 | 18,403 | NA |
| Averted by standard vaccine | 50% |  | 2,943 | 691 | 2,391 | NA |
| Incremental burden averted by improved vaccine | 50% | 5% | 180 | 231 | 400 | NA |
|  |  | 10% | 360 | 463 | 801 | NA |
|  |  | 15% | 540 | 694 | 1,201 | NA |
|  |  | 20% | 719 | 925 | 1,601 | NA |
|  |  | 25% | 899 | 1,156 | 2,001 | NA |
|  |  | 30% | 1,079 | 1,388 | 2,402 | NA |
|  |  | 35% | 1,259 | 1,619 | 2,802 | NA |
|  |  | 40% | 1,439 | 1,850 | 3,202 | NA |
| **Influenza-associated P&I hospitalisations^b^** | | | | | | |
| Standard vaccine, Baseline | As per Table 1 |  | 8,768 | 7,789 | 14,700 | NA |
| No vaccination | 0% |  | 9,863 | 8,060 | 15,736 | NA |
| Standard vaccine | 50% |  | 7,644 | 7,536 | 13,927 | NA |
| Averted by standard vaccine | 50% |  | 2,219 | 524 | 1,810 | NA |
| Incremental burden averted by improved vaccine | 50% | 5% | 136 | 175 | 303 | NA |
|  |  | 10% | 271 | 351 | 606 | NA |
|  |  | 15% | 407 | 526 | 909 | NA |
|  |  | 20% | 542 | 701 | 1,212 | NA |
|  |  | 25% | 678 | 877 | 1,515 | NA |
|  |  | 30% | 814 | 1,052 | 1,818 | NA |
|  |  | 35% | 949 | 1,227 | 2,120 | NA |
|  |  | 40% | 1,085 | 1,402 | 2,423 | NA |
| **Influenza-associated P&I deaths^b^** | | | | | | |
| Standard vaccine, Baseline | As per Table 1 |  | 37 | 51 | 156 | 32 |
| No vaccination | 0% |  | 41 | 53 | 167 | 39 |
| Standard vaccine | 50% |  | 32 | 49 | 148 | 29 |
| Averted by standard vaccine | 50% |  | 9 | 3 | 19 | 10 |
| Incremental burden averted by improved vaccine | 50% | 5% | 0.6 | 1.1 | 3.2 | 0.5 |
|  |  | 10% | 1.1 | 2.3 | 6.4 | 0.9 |
|  |  | 15% | 1.7 | 3.4 | 9.7 | 1.4 |
|  |  | 20% | 2.3 | 4.6 | 12.9 | 1.9 |
|  |  | 25% | 2.9 | 5.7 | 16.1 | 2.3 |
|  |  | 30% | 3.4 | 6.9 | 19.3 | 2.8 |
|  |  | 35% | 4.0 | 8.0 | 22.5 | 3.3 |
|  |  | 40% | 4.6 | 9.2 | 25.8 | 3.7 |

^a^ rVE indicates the relative vaccine effectiveness of the improved compared to the standard vaccine,

^b^ Estimates based on modelled influenza-associated hospitalisations^2^ and deaths^3^. Data not available for 2019.

### Supplementary References

1. Australian Government Department of Health and Aged Care. National Notifiable Diseases Surveillance System (NNDSS) public datasets: Influenza (laboratory confirmed) notifications in Australia Canberra: Australian Government; 2023 [updated 2023; cited 2023 August 7]. Available from: <https://www.health.gov.au/resources/collections/nndss-public-datasets?utm_source=health.gov.au&utm_medium=callout-auto-custom&utm_campaign=digital_transformation>.

2. Nazareno AL, Muscatello DJ, Turner RM, Wood JG, Moore HC, Newall AT. Modelled estimates of hospitalisations attributable to respiratory syncytial virus and influenza in Australia, 2009-2017. Influenza Other Respir Viruses. 2022;16(6):1082-90.

3. Muscatello DJ, Nazareno AL, Turner RM, Newall AT. Influenza-associated mortality in Australia, 2010 through 2019: High modelled estimates in 2017. Vaccine. 2021;39(52):7578-83.

4. Cheng AC, Holmes M, Dwyer DE, Irving LB, Korman TM, Senenayake S, et al. Influenza epidemiology in patients admitted to sentinel Australian hospitals in 2015: the Influenza Complications Alert Network. Commun Dis Intell Q Rep. 2016;40(4):E521-e6.

5. Cheng AC, Holmes M, Dwyer DE, Irving L, Korman T, Senenayake S, et al. Influenza epidemiology in patients admitted to sentinel Australian hospitals in 2016: the Influenza Complications Alert Network (FluCAN). Commun Dis Intell Q Rep. 2017;41(4):E337-e47.

6. Cheng AC, Holmes M, Dwyer DE, Senanayake S, Cooley L, Irving LB, et al. Influenza epidemiology in patients admitted to sentinel Australian hospitals in 2017: the Influenza Complications Alert Network (FluCAN). Commun Dis Intell (2018). 2019;43.

7. Cheng A, Holmes M, Dwyer DS, S Cooley, L , Irving L, Simpson GK, T, Macartney K, et al. Vaccine effectiveness in patients admitted to sentinel Australian hospitals in 2018: the Influenza Complications Alert Network (FluCAN). 2018.

8. Cheng AC, Dwyer DE, Holmes M, Irving L, Simpson G, Senenayake S, et al. Influenza epidemiology in patients admitted to sentinel Australian hospitals in 2019: the Influenza Complications Alert Network (FluCAN). Commun Dis Intell (2018). 2022;46.

9. Cheng AC, Holmes M, Dwyer DE, Senanayake S, Cooley L, Irving LB, et al. Influenza epidemiology in patients admitted to sentinel Australian hospitals in 2018: the Influenza Complications Alert Network (FluCAN). Commun Dis Intell (2018). 2019;43.
